# Prediction of Protein Modification Sites of Pyrrolidone Carboxylic Acid Using mRMR Feature Selection and Analysis

**DOI:** 10.1371/journal.pone.0028221

**Published:** 2011-12-09

**Authors:** Lu-Lu Zheng, Shen Niu, Pei Hao, KaiYan Feng, Yu-Dong Cai, Yixue Li

**Affiliations:** 1 Hubei Bioinformatics and Molecular Imaging Key Laboratory, Huazhong University of Science and Technology, Wuhan, China; 2 Shanghai Center for Bioinformation Technology, Shanghai, China; 3 Key Laboratory of Systems Biology, Shanghai Institutes for Biological Sciences, Chinese Academy of Sciences, Shanghai, China; 4 Institute of Systems Biology, Shanghai University, Shanghai, China; University of South Florida College of Medicine, United States of America

## Abstract

Pyrrolidone carboxylic acid (PCA) is formed during a common post-translational modification (PTM) of extracellular and multi-pass membrane proteins. In this study, we developed a new predictor to predict the modification sites of PCA based on maximum relevance minimum redundancy (mRMR) and incremental feature selection (IFS). We incorporated 727 features that belonged to 7 kinds of protein properties to predict the modification sites, including sequence conservation, residual disorder, amino acid factor, secondary structure and solvent accessibility, gain/loss of amino acid during evolution, propensity of amino acid to be conserved at protein-protein interface and protein surface, and deviation of side chain carbon atom number. Among these 727 features, 244 features were selected by mRMR and IFS as the optimized features for the prediction, with which the prediction model achieved a maximum of MCC of 0.7812. Feature analysis showed that all feature types contributed to the modification process. Further site-specific feature analysis showed that the features derived from PCA's surrounding sites contributed more to the determination of PCA sites than other sites. The detailed feature analysis in this paper might provide important clues for understanding the mechanism of the PCA formation and guide relevant experimental validations.

## Introduction

Post-translational modifications (PTMs) are crucial for proteins to maintain their structural and functional diversities in both prokaryotes and eukaryotes. They influence a protein's state of activity, localization, turnover and ability to interact with other molecules [1], which are pivotal for many cellular processes, e.g. in signal transduction, kinase induced cascades are turned off and on by the removals or additions of phosphate groups in proteins [2]. Pyrrolidone carboxylic acid (PCA), also known as pyroglutamic acid or pGlu, is produced during one of the common PTMs, and may be formed naturally by an enzymatic synthesis from N-terminal glutamine under mildly acid conditions or as an artifact from N-terminal glutamic acid under very acid conditions in proteins or peptides [3], [4]. As a glutamic acid derivative that lacks a H_2_O molecule [3] in extracellular and multi-pass membrane proteins, many studies have demonstrated that pyroglutamic acid is formed either late in protein translation by cyclization of the glutamine at the N-terminus or as a post-translational event, just prior to the secretion of completed proteins from the cell [5]. Modified proteins of this type usually show an increase half-life, because PCA blocks proteins, minimizing their susceptibility to degradation by aminopeptidases [3], [6]. Structures containing the cyclic product of glutamine at the N-terminus are common in nature [3]. In general, those proteins or peptides have an endocrine and/or regulative function in mammalian tissues and are of high interest, since their concentrations in the blood could influence synthesis pathways of important metabolites [7]. A particularly well-studied example is the tripeptide thyrotropin releasing factor (TRF), which has the sequence pGlu-His-Pro that stimulates the release of thyrotropin in vivo [8] and has been shown to be able to enhance prolactin synthesis and decrease growth hormone production in vitro [5], [9]. Hinkle and Tashjian have proved that any structural substitution in the pGlu lactam ring of TRF will significantly decrease both hormone synthesis and receptor binding ability [8]. Aside from functions as an incorporated amino acid, functions of free pyrrolidone carboxylic acid are less clear, though its pharmacological properties have been described repeatedly [6]. It has been shown that pGlu stimulates GABA releasing from the cerebral cortex and therefore produces anti-anxiety effects in a simple approach-avoidance conflict situation in the rat [10]. In addition, Silva et al. have confirmed that L-pyroglutamic acid predominantly accumulates in the inherited metabolic diseases, glutathione synthetase deficiency (GSD) and γ-glutamylcysteine synthetase deficiency (GCSD), by reducing brain CO2 production, lipid biosynthesis and ATP levels [11]. High anion gap and metabolic acidosis in adults is also proved to associate with accumulation of pGlu [12]. Thus, identification of protein pyroglutamic acid modification sites is of fundamental importance to understand the mechanism by which pGlu occurs in biological systems.

So far, the identification of pGlu modification sites has largely been focused on the mass spectrometry (MS) technique [13]–[16], which measures mass-to-charge ratio (*m/z*), yielding the molecular mass and the fragmentation pattern of peptides derived from proteins. Owing to its high-throughput and accuracy, MS technique represents a gold method for all modifications that change the molecular weight. Based on MS data, Wilkins et al. have developed a software tool, FindMod, to discover 22 post-translational modifications, including acetylation, phosphorylation and pyrrolidone carboxylic acid and so on [13]. They examined 5153 incidences of post-translational modifications annotated in the SWISS-PROT database, and made a total of 29 rules to predict the amino acids in the peptide that might carry the modification. For the PCA modification, they used the rules with monoisotopic and average delta masses being −17.0266 and −17.0306, the modified amino acid being Q and the position of amino acid in proteins being at the N-terminus. However, this MS method is time-consuming and costly. Developing novel methods *in silico* that merely depend on amino acid sequences is urgent, especially for large scale proteomic analysis.

dbPTM is a database that contains information about protein post-translational modifications (PTMs), such as modified sites, solvent accessibility of amino acid residues, protein secondary and tertiary structures, protein domains and protein variations [17]. From the database, we found that 689 experimentally validated residues modified to be pyrrolidone carboxylic are amino acid Glns (Q), and only 2 are amino acid Glus (E). This seems to illustrate that pyrrolidone carboxylic acid derived from a glutamate residue is extremely rare and may be correlated with an extreme acidic context that the protein involved, and also explain why FindMod used the rule that the amino acid pGlu modified is Q exclusively. dbPTM also indicates that the Q residue carrying the modification with surrounding sites usually locates in a coiled secondary structure, but no specific sequence conservation patterns can be observed in these residues, while sites around E residue are restricted to LTGERL. Moreover, based on kinasePhos-like method, which is to computationally predict phosphorylation sites by applying profile hidden markov model (HMM) [18], dbPTM predicted 12,322 PCA sites using protein sequences taken from Swiss-Prot. However, we cannot get enough detailed information to compare this method with others. We also noticed that the number of predicted sites is much greater than that of experimentally validated sites, implying the predictor does not perform well or many PCA modification sites have not yet be identified by experimental methods.

In this paper, we present a new computational method to predict modification sites of PCA in protein sequences. Nearest neighbor algorithm (NNA), a kind of machine learning approach, incorporated by feature selection (IFS based on mRMR), was applied to make predictions. The features used in the method come from many different sources, and can be grouped into 7 categories: position-specific conversation scoring matrixes (PSSM), amino acid factors, disorder scores, secondary structure and solvent accessibility, gain/loss of amino acids during evolution, propensity of amino acid to be conserved at protein-protein interface and protein surface, and deviation of side chain carbon atom number. Our method achieved an overall MCC of 78.12% using the optimal feature set. Feature analysis shows that the conservation of amino acids at some certain residues in the upstream of PCA modification sites plays more important roles in the prediction; it also shows that the remaining features of amino acids in the flanking regions are important for the prediction.

## Materials and Methods

### Dataset

We downloaded 1366 protein sequences containing modification sites of PCA from uniprot (version 2011_02) [19], [20]. These protein sequences totally contain 1528 modification sites of PCA, within which we selected 769 sites annotated as “Note = Pyrrolidone carboxylic acid” for further analysis. These 769 sites include 370 sites located in the internal region and 399 sites located at the N-terminal of the relevant proteins. We only considered the 370 sites located in the internal region of the 299 protein sequences.

We extracted one 21-residue peptide fragment for each PCA site with the modification site of PCA at the centre, plus 10 residues upstream and 10 residues downstream of the modification site. The peptides with length less than 21 residues were complemented by character “-”. After removing identical peptide fragments, there remain 333 fragments.

For negative samples, we extracted totally 3635 Q sites located in the internal region of the 299 protein sequences. Then we removed Q sites identical to positive samples or within the 3635 Q sites themselves, resulting in totally 2997 peptide fragments, from which we randomly selected 1665 (333*5 = 1665) peptide fragments as the negative samples. Thus, there were totally 1998 samples, including 333 positive samples and 1665 negative samples. Both positive and negative samples and their surrounding amino acids were given in Dataset S1.

### Feature Construction

#### The features of PSSM conservation scores

Evolutionary conservation plays important roles in biological analysis. A more conserved residue within a protein sequence usually indicate that it is more important for protein functioning and thus under stronger selective pressure.

We used Position Specific Iterative BLAST (PSI BLAST) [21] to measure the conservation status for a specific residue. It used a 20-dimensional vector to denote probabilities of conservation against mutations to 20 different amino acids for a specific residue. For a given peptide, all such 20-dimentional vectors for all residues composed a matrix called position specific scoring matrix (PSSM). More conserved residues through cycles of PSI BLAST were suggested to be more important for biological functioning.

In this study, we used PSSM conservation score to quantify the conservation status of each amino acid in a protein sequence.

#### The features of amino acid factors

Since each of the 20 amino acids has various and specific properties, the composition of different residues within a protein can influence the specificity and diversity of the protein structure and function. AAIndex [22] is a database containing various physicochemical and biochemical properties of amino acids. Atchley et al. [23] performed multivariate statistical analyses on AAIndex and transformed AAIndex to five multidimensional and highly interpretable numeric patterns of attribute covariation reflecting polarity, secondary structure, molecular volume, codon diversity, and electrostatic charge. These five numerical pattern scores (denoted as “amino acid factors”) have been applied successfully in many researches [24]–[26]. We also used the amino acid factors to represent the respective properties of each amino acid in a given protein.

#### The features of disorder score

Protein segments lacking fixed three-dimensional structures under physiological conditions play important roles in biological functions [27], [28]. The disordered regions of proteins allow for more modification sites and interaction partners and always contain PTM sites, sorting signals, and protein ligands. Thus it is quite importance for protein structure and function [27], [29], [30]. In this study, VSL2 [31], which can accurately predict both long and short disordered regions in proteins, was used to calculate disorder score that denotes the disorder status of each amino acid in a given protein sequence.

#### The features of secondary structure and solvent accessibility

Protein structures play important roles in protein functioning and the post-translational modification of specific residues may be influenced by the solvent accessibility of the relevant residues. So we also considered protein structures including secondary structure and solvent accessibility to encode each peptide. These features were predicted by SSpro 4 [32], which classify secondary structure of each amino acid as ‘helix’, ‘strand’, or ‘other’, and solvent accessibility as ‘buried’ or ‘exposed’. The usage of the secondary structure feature predicted by SSpro4 in our study could indicate whether a specific type of these three secondary structure types is associated with protein PCA modification sites.

#### Gain/loss of amino acids during evolution,propensity of amino acid to be conserved at protein-protein interface and protein surface

It has been suggested by Goldsmidt et al that protein folding has evolved to remove regions of high propensity and remain proper conformation for fibrillation from protein surfaces [33]. We included features of gain/loss of amino acids during evolution [34] in our analysis. Since the location of a residue on protein surface or interaction interface may also influence the determination of PCA modification site, we also included the features of conservation of an amino acid on protein-protein interaction interface and protein exposed surface [35].

#### Deviation of side chain carbon atom number

Different atoms may have their various intrinsic properties [36]. Different composition of atoms within a residue or peptide could also influence protein properties and thus influence protein structures and functions. So, we calculated the deviation of side chain carbon atom number for each residue within the 21-residue segment. This feature was calculated by subtracting the mean carbon atom number of side chains within a 21-residue segment by the side chain carbon number of each residue.

#### The feature space

Since the residue at site 11 of the 21-peptide is Q, so for this site we incorporated 27 features, including 20 features of PSSM conservation score, 1 feature of disorder score, 3 features of secondary structure, 2 features of solvent accessibility and 1 feature of deviation of side chain carbon atom number. For other residues, we incorporated totally 35 features by adding other 8 features, including 5 features of AAFactor, 2 features of propensity of amino acid to be conserved at protein-protein interface and protein surface and 1 feature of gain/loss of amino acids during evolution. Overall for the 21-residue protein segment, there are totally 35*20+27 = 727 features.

For residues denoted by “-”, the carbon atom number of side chains was set to be the mean of the side chain carbon atom numbers of the 20 amino acids, and other features were set to be 0.

### mRMR method

We used Maximum Relevance Minimum Redundancy (mRMR) method to rank the importance of the 727 features [37]. mRMR method could rank features based on both their relevance to the target and the redundancy of features. A smaller index of a feature denotes that it has a better trade-off between maximum relevance to target and minimum redundancy.

Both relevance and redundancy was quantified by mutual information (MI), which estimates how much one vector is related to another. The MI equation was defined as below:
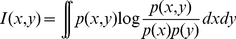
(1)In equation (1),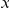, 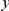 are vectors, 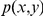 is their joint probabilistic density, and 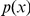 and 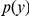 are the marginal probabilistic densities.


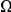
 was used to denote the whole feature set. 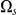 was used to denote the already-selected feature set containing m features and 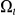 was used to denote the to-be-selected feature set containing n features. The relevance 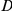 between the feature 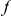 in 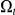 and the target 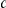 can be calculated by:
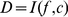
(2)The redundancy 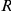 between the feature 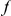
in 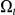 and all the features in 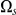 can be calculated by:
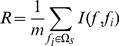
(3)To get the feature 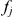 in 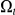 with maximum relevance and minimum redundancy, the mRMR function combined equation (2) and equation (3) and are defined as below:
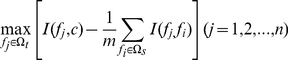
(4)The mRMR feature evaluation would be run N rounds when given a feature set with N (N = m+n) features. After the evaluation, we get an ordered feature set 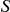:
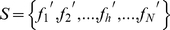
(5)In 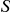, index h of a feature indicates at which round that the feature is selected. The smaller the index h is, the earlier the feature satisfied equation (4) and the better the feature is.

### Nearest Neighbor Algorithm

Nearest Neighbor Algorithm (NNA) was used to predict PCA modification sites. NNA calculates the similarities between the test sample and all the training samples and by which makes its classification decision. In our study, the distance between vector 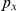 and 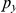 is defined as below [38], [39]:
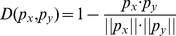
(6)In equation (6), 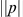 is the module of vector 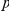. 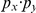 denotes the inner product of 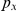 and 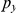. The smaller 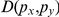, the more similar 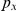 to 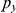 is.

In NNA, given a training set 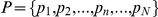 and a vector 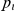, 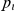 will be designated to the same class of its nearest neighbor 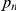 in 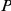, which is the vector having the smallest 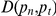:
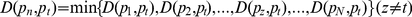
(7)


### Jackknife Cross-Validation Method

Jackknife Cross-Validation Method [40]–[42] (also called the Leave-one-out cross-validation, LOOCV) was used to evaluate the performance of a classifier. In Jackknife Cross-Validation Method, every sample is tested by the predictor that is trained with all the other samples. Let TP denotes true positive. TN denotes true negative. FP denotes false positive and FN denotes false negative. To evaluate the performance of our PCA modification site predictor, the prediction accuracy, specificity, sensitivity and MCC (Matthews's correlation coefficient) were calculated as below:
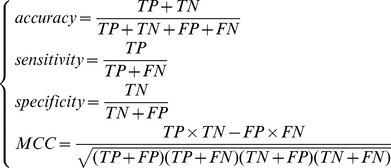
(8)


### Incremental Feature Selection (IFS)

Based on the ranked features derived from mRMR, we used Incremental Feature Selection (IFS) [39], [43] to determine the optimal number of features.

During IFS procedure, features in the ranked feature set are added one by one from higher to lower rank. A new feature set is composed when one feature is added. Thus given N ranked features, N feature sets would be composed. The i-th feature set is:
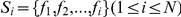
For each of the N feature sets, an NNA predictor was constructed and tested using Jackknife cross-validation test. With N prediction accuracy, sensitivity, specificity and MCC calculated, we obtain an IFS table with one column being the index i and the other columns to be the prediction accuracy, sensitivity, specificity and MCC. We then can get the optimal feature set (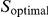), with which the predictor achieves the best prediction performance.

## Results

### mRMR result

We get the ranked mRMR feature list of 727 features using mRMR method. A smaller index of a feature suggests that it is more important for the prediction of PCA modification site. This ranked 727-feature list was used in IFS procedure for the selection of optimal feature set.

### IFS result

**Figure 1:**
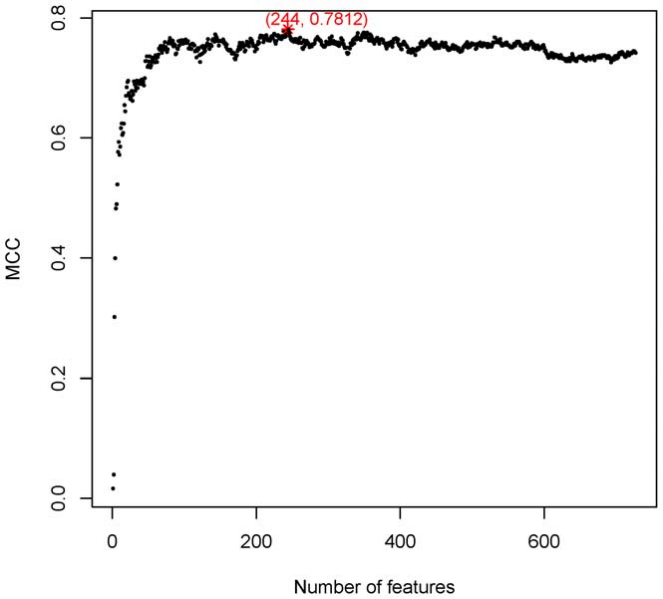
Distribution of MCC values against feature numbers. IFS predictive MCC values were plotted against feature numbers based on Table S1. The maximum MCC is 0.7812 using 244 features. These 244 features were considered as the optimal feature set of our classifier.

By adding the ranked features one by one, we built 727 individual predictors for the 727 sub-feature sets to predict PCA modification sites. We then tested the prediction performance of each of the 727 predictors and get the IFS results (given in Table S1). Figure 1 shows the IFS curve plotted based on Table S1. The maximum MCC is 0.7812 when 244 features are included. These 244 features were considered as the optimal feature set of our classifier. Using which, the predictive sensitivity, specificity and accuracy were 0.8523, 0.9528, and 0.9355 respectively. The 244 optimal features were given in Table S2 and the top 20 features in Table 1.

**Table 1:** Top 20 features of the optimal feature set for pyrrolidone acid modification sites.

Order	Name	Site	Feature
1	AA10-Pssm_A	10	PSSM
2	AA1-Pssm_L	1	PSSM
3	AA13-Pssm_W	13	PSSM
4	AA2-Codon Diversity	2	AAFactor
5	AA4-Pssm_L	4	PSSM
6	AA10-Pssm_N	10	PSSM
7	AA3-Pssm_L	3	PSSM
8	AA8-Pssm_A	8	PSSM
9	AA5-Polarity	5	AAFactor
10	AA10-Codon Diversity	10	AAFactor
11	AA1-Secondary Structure Helix	1	Secondary structure
12	AA8-Pssm_C	8	PSSM
13	AA10-Pssm_I	10	PSSM
14	AA9-Pssm_I	9	PSSM
15	AA13-Secondary Structure Other	13	Secondary structure
16	AA1-Pssm_N	1	PSSM
17	AA5-Pssm_A	5	PSSM
18	AA6-Pssm_Q	6	PSSM
19	AA8-Side Chain Count of Atom_C Deviation from Mean	8	Deviation of side chain carbon atom number
20	AA2-Pssm_L	2	PSSM

### Feature analysis of optimal feature set

**Figure 2:**
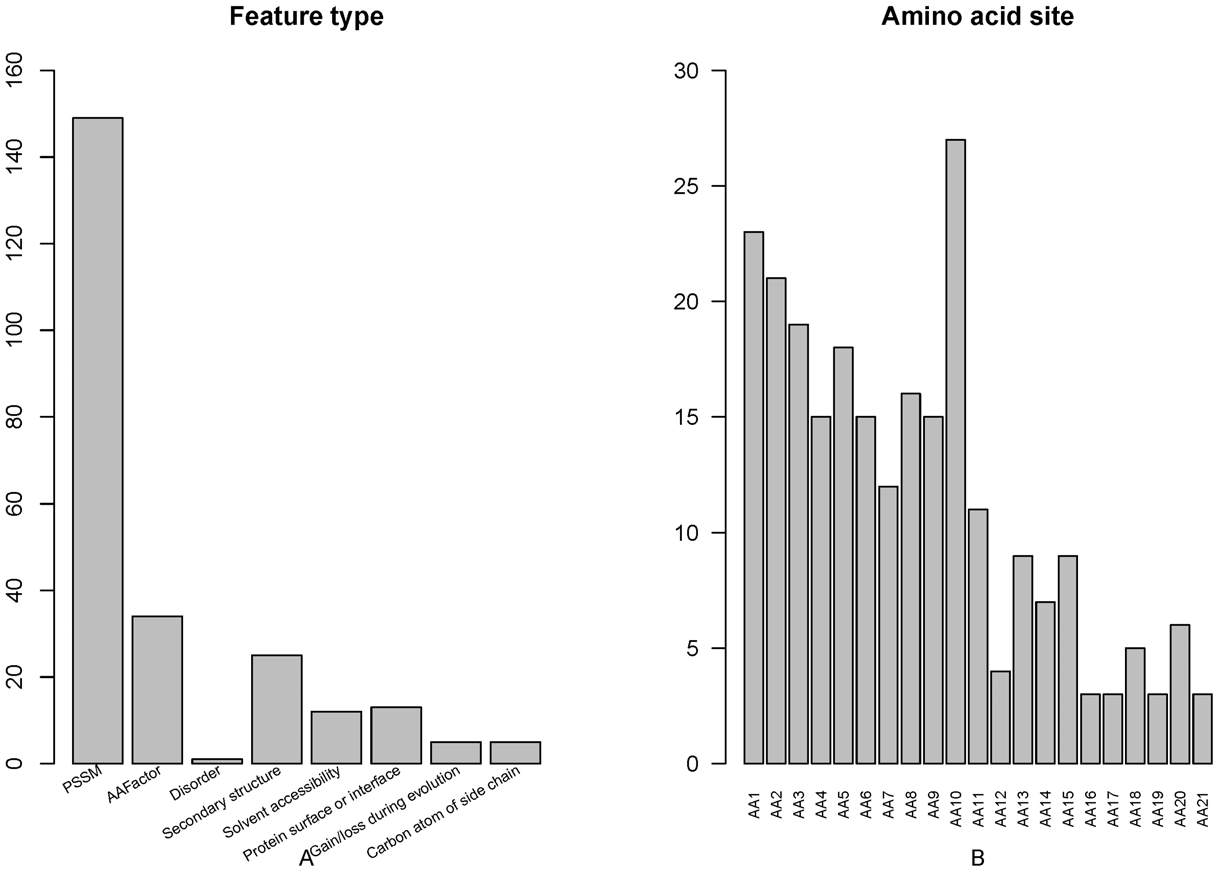
Feature and site specific distribution of the optimal feature set. (A) Feature distribution of the optimal feature set. Among the optimized 244 features, there were 149 features of PSSM conservation score, 34 features of amino acid factor, 1 feature of disorder, 25 features of secondary structure, 12 features of solvent accessibility, 13 features of propensity on protein surface or protein-protein-interaction interface (“Protein surface or interface”), 5 features of gain/loss during evolution and 5 features of deviation of side chain carbon atom number (“Carbon atom of side chain”). (B) Site specific distribution of the optimal feature set. The site-specific distribution of the optimal feature set revealed that site 10 played the most important role in the prediction of PCA modification. Site 1, 2, 3 and 5 also played relatively more role than the remaining sites.

The distribution of the number of each type of features in the optimal feature set was investigated and shown in Figure 2A. Among the optimized 244 features, there were 149 features of PSSM conservation score, 34 features of amino acid factor, 1 feature of disorder, 25 features of secondary structure, 12 features of accessibility, 13 features of propensity on surface or interface, 5 features of gain/loss during evolution and 5 features of deviation of side chain carbon atom number.

The site-specific distribution of the optimal feature set (Figure 2B) revealed that site 10 played the most important role in the determination of PCA modification sites. And site 1, 2, 3 and 5 played more important role than the remaining sites.

### Feature analysis of PSSM conservation score

**Figure 3:**
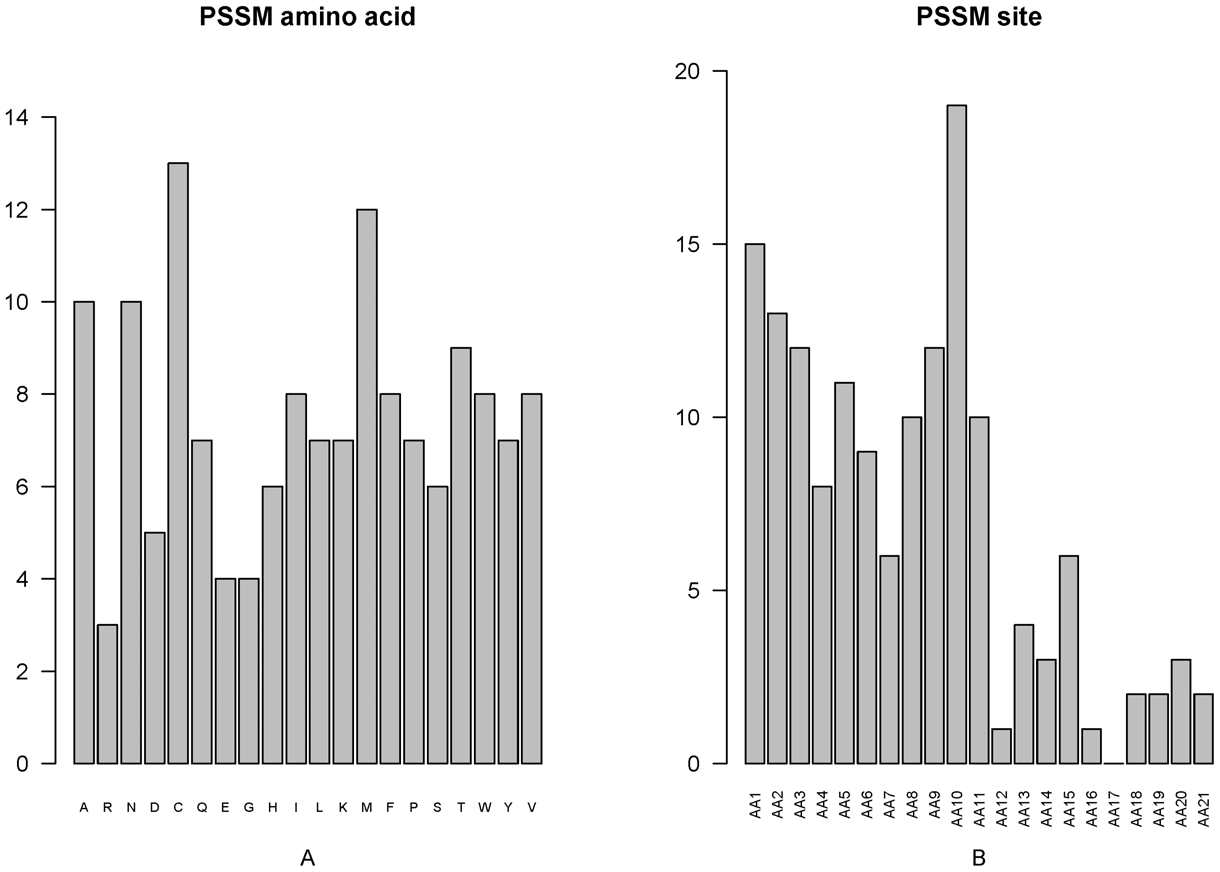
Feature and site specific distribution of the PSSM features in the optimal feature set. (A) Feature distribution of the PSSM features in the optimal feature set. The conservation against mutations to amino acid C, M A, N and T influent more on PCA modification determination than the mutations to other amino acids. (B) Site-specific distribution of the PSSM features in the optimal feature set. The conservation status of “AA10”, “AA1” and “AA2” sites were most important for the PCA modification site prediction.

Among the optimized 244 features, there were 149 features of PSSM conservation score, the greatest proportion of the optimized features. We investigated the number of each kind of amino acids of the PSSM features (Figure 3A) and found that the conservation against mutations to the 20 amino acids influents differently on the prediction of PCA modification site. Mutations to amino acid C, M A, N and T influence more on PCA modification determination than the mutations to other amino acids. We also investigated the number of PSSM features at each site (Figure 3B). The conservation status of “AA10”, “AA1” and “AA2” sites were most important for the prediction of PCA modification site, as shown in Figure 3B.

### Feature analysis of amino acid factor

**Figure 4:**
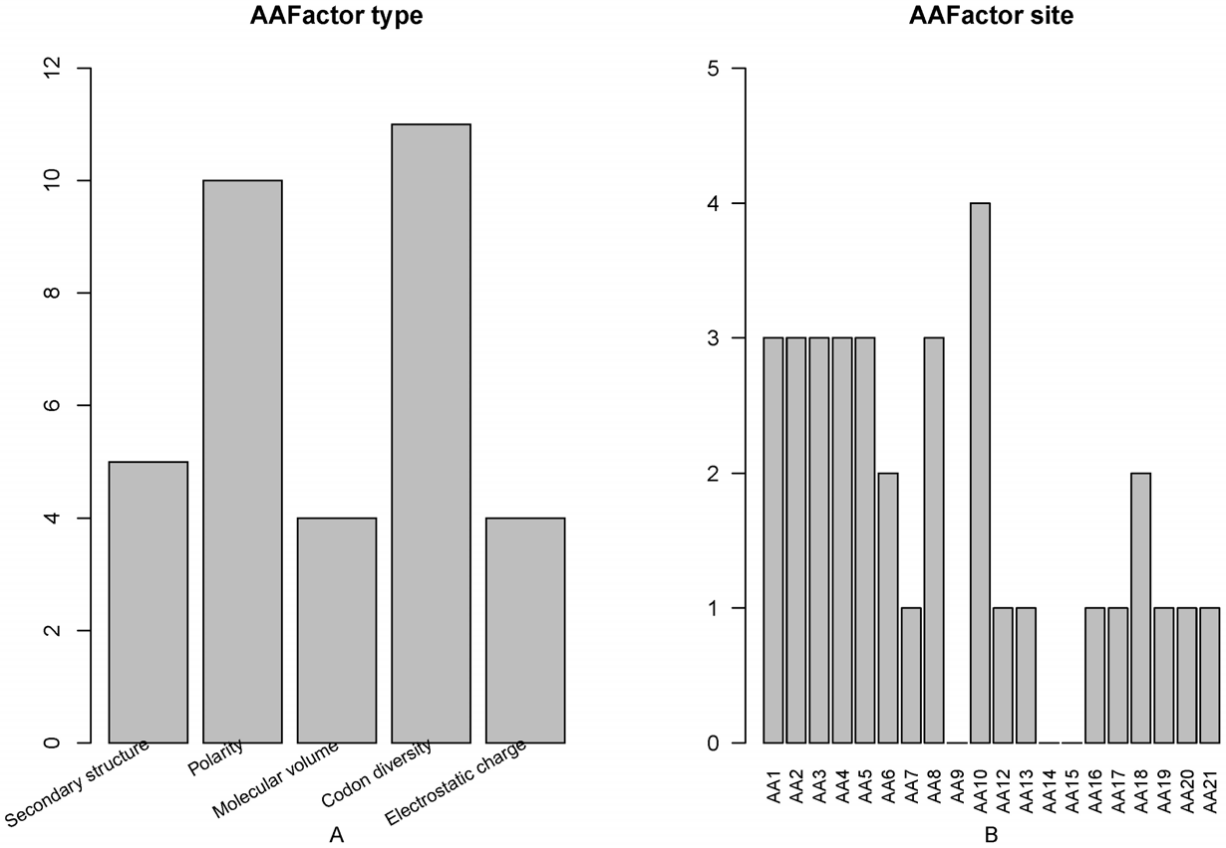
Feature and site specific distribution of the amino acid factor features in the optimal feature set. (A) Feature distribution of the amino acid factor features in the optimal feature set. Codon diversity and polarity features were the most important features, and secondary structure, molecular volume and electrostatic charge were almost equally important features to the PCA modification site prediction. (B) Site-specific distribution of the amino acid factor features in the optimal feature set. Residues at site 10 have the most important effect on PCA modification site prediction. Residues at site 1–5 and site 8 had relatively more effect on PCA modification site prediction.

The number of each type of amino acid factor features (Figure 4A) and the number of amino acid factor features at each site (Figure 4B) were analyzed. It was found that codon diversity and polarity were the most important features, and secondary structure, molecular volume and electrostatic charge were almost equally important features. In Figure 4B, residues at site 10 contribute most to the prediction of PCA modification site, followed by residues at site 1–5 and site 8 which contribute less, and then the residues at the remaining sites which contribute least to the prediction.

### Feature analysis of disorder score

Among the optimal feature set, only 1 disorder feature was selected, the disorder feature at site 14, having an index of 77.

### Feature analysis of secondary structure and solvent accessibility

**Figure 5:**
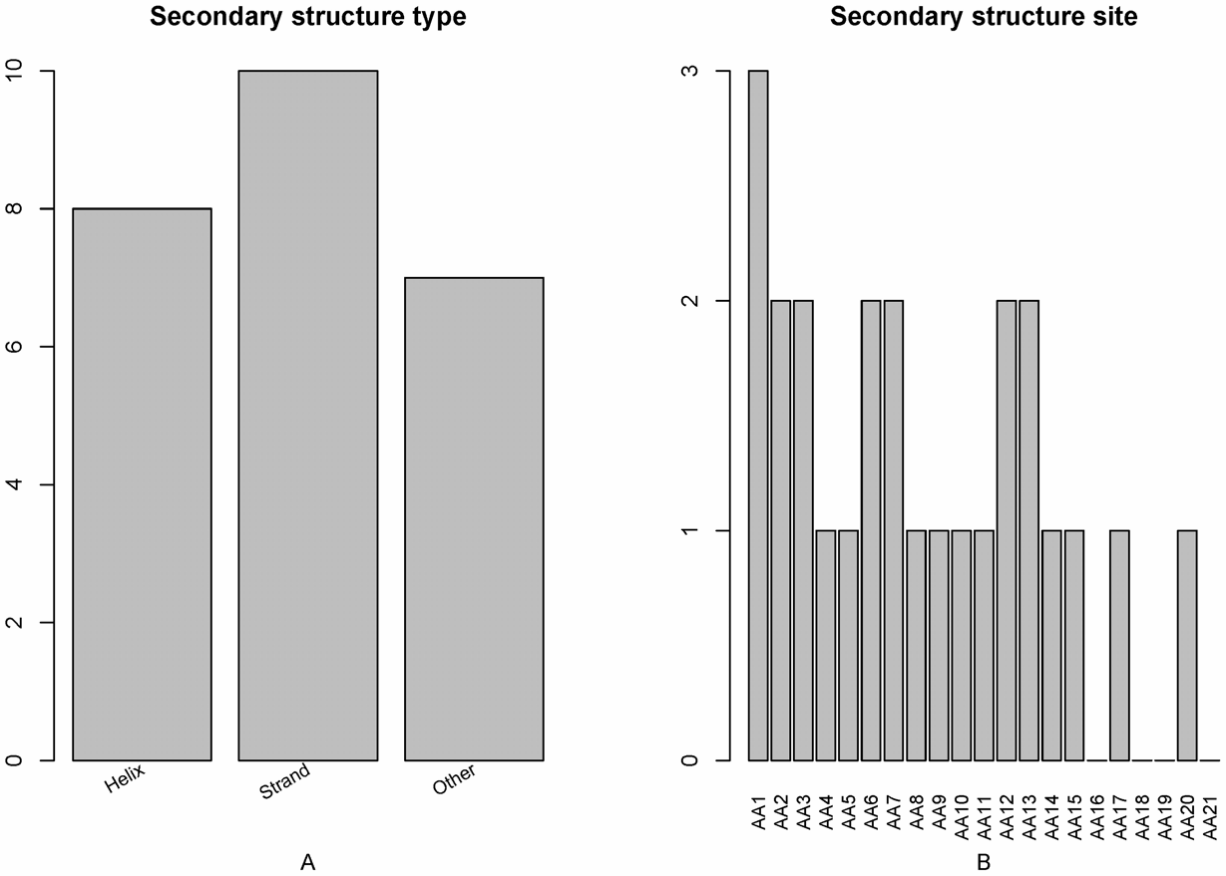
Feature and site specific distribution of the secondary structure features in the optimal feature set. (A) Feature distribution of the secondary structure features in the optimal feature set. All the three types of secondary structure features (helix, strand and other) can influence the PCA modification site determination. (B) Site-specific distribution of the secondary structure features in the optimal feature set. The secondary structure of site 1 may influence more to PCA modification site determination than other sites. The secondary structure features at site 2, 3, 6, 7, 12 and 13 may also influence more on PCA site determination.

The feature- and site-specific distribution of the secondary structures in the optimal feature set was shown in Figure 5. From Figure 5A, we can see that all three types of secondary structures (helix, strand and other) influence the PCA modification site determination. From 5B, we can see that secondary structure of site 1 may influence more to the determination of PCA modification site than other sites. The secondary structures at site 2, 3, 6, 7, 12 and 13 may also influence more on PCA site determination.

**Figure 6:**
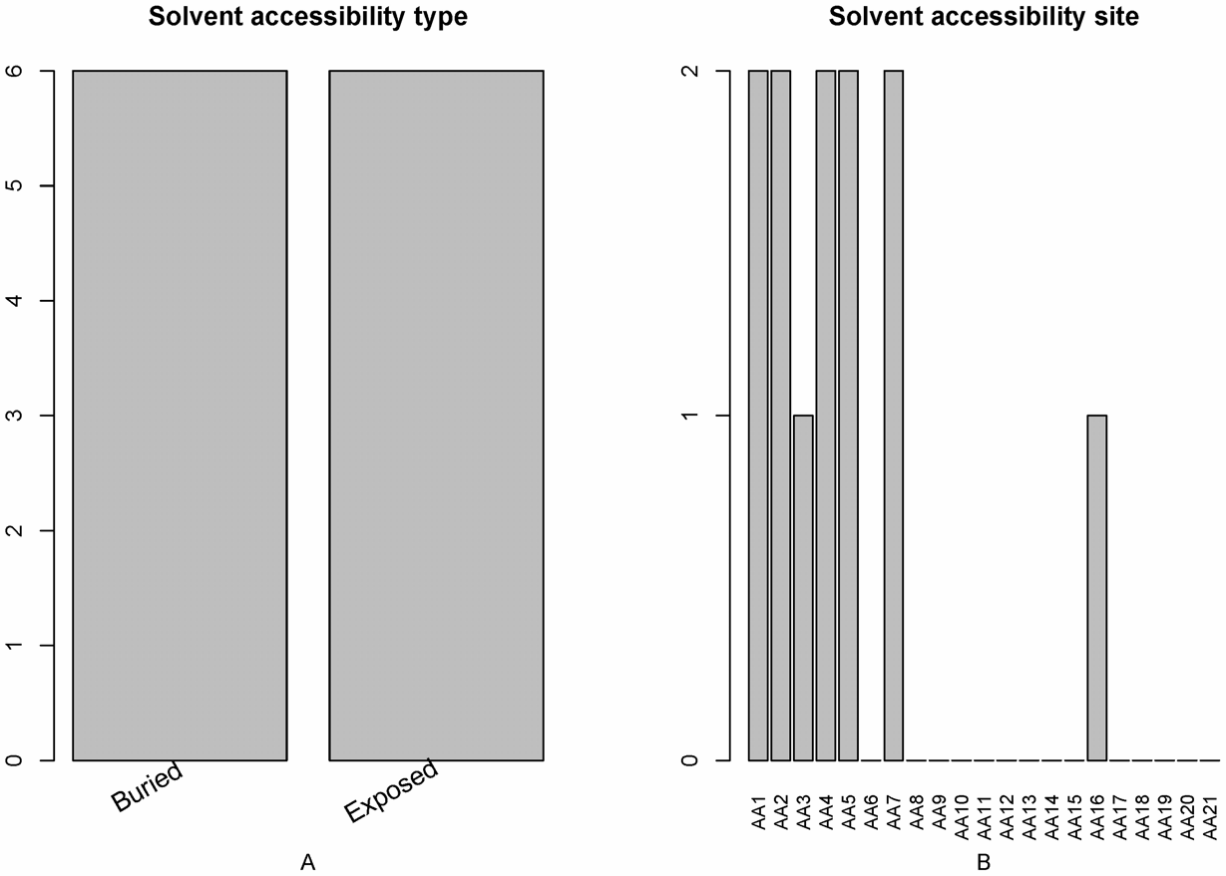
Feature and site specific distribution of the solvent accessibility features in the optimal feature set. (A) Feature distribution of the solvent accessibility features in the optimal feature set. The number of the two types of solvent accessibility features (buried and exposed) is equal (6), indicating that features derived from solvent accessibility are equally important for PCA site determination. (B) Site-specific distribution of the solvent accessibility features in the optimal feature set. Solvent accessibility features at site 1, 2, 4, 5, 7, 3 and 16 influences more on PCA site determination.

We also investigated the 12 features of solvent accessibility in the optimal feature set (Figure 6). As shown in Figure 6A, the number of the two types of solvent accessibility features (buried and exposed) is equal (6) to each other, indicating that both types of solvent accessibility are important for PCA site determination. Figure 6B showed that solvent accessibility features at site 1, 2, 4, 5, 7 3 and 16 influence more on PCA site determination.

### Features analysis of propensity of amino acid to be conserved at protein-protein interface and protein surface

**Figure 7:**
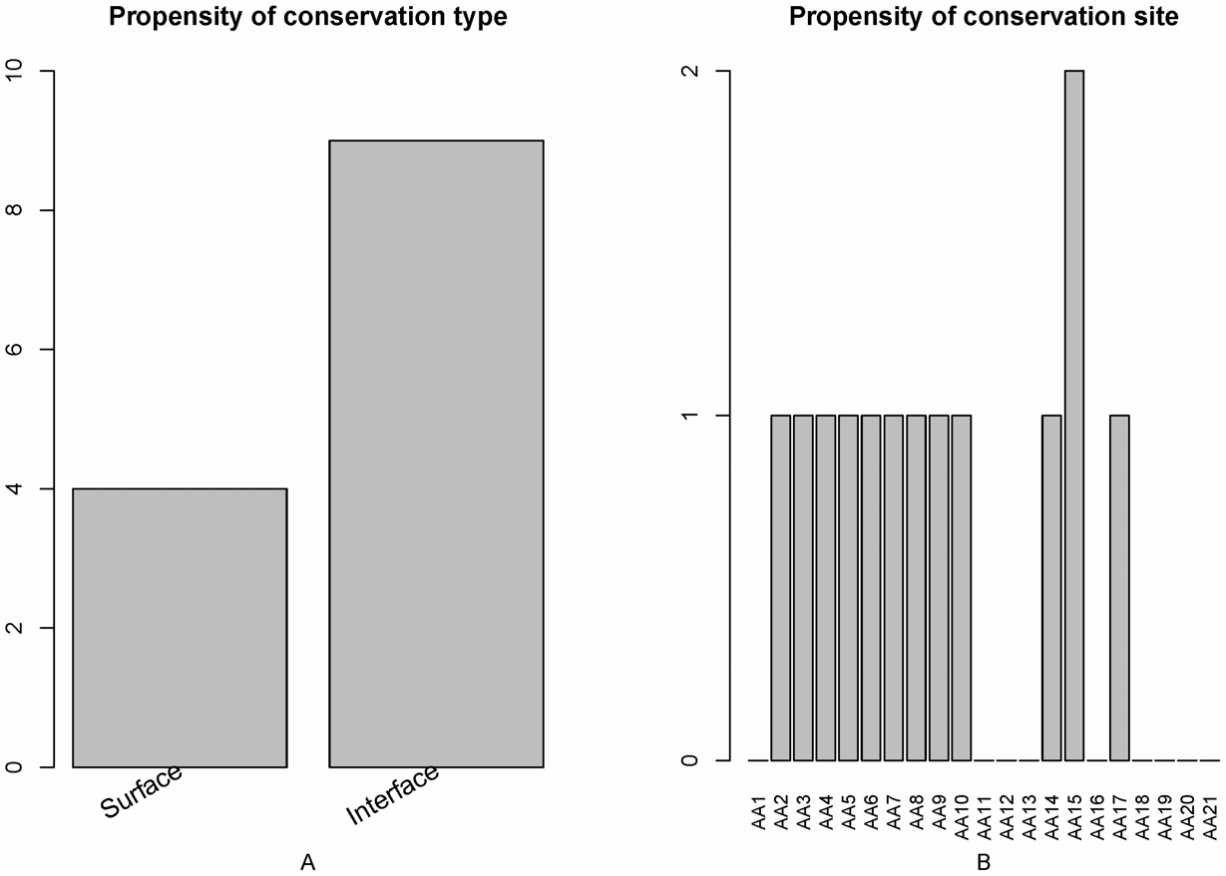
Feature and site specific distribution of the propensity of amino acid to be conserved at protein-protein interface and protein surface features in the optimal feature set. (A) Feature distribution of these features in the optimal feature set. Propensity of amino acid to be conserved at protein-protein interface influence more on PCA modification site determination and propensity of amino acid to be conserved at protein surface also can influence PCA modification site determination. (B) Site-specific distribution of these features in the optimal feature set. Propensity of amino acid to be conserved at protein-protein interface and protein surface features at site 15, 2–10, 14 and 17 influence more on PCA modification site determination.

There were 13 features of propensity of amino acid to be conserved at protein-protein interface and protein surface in the optimal feature set. As shown in Figure 7A, propensity of amino acid to be conserved at protein-protein interface influence more on PCA modification site determination and propensity of amino acid to be conserved at protein surface also influence PCA modification site determination.

As shown in Figure 7B, the Propensity of amino acid to be conserved at protein-protein interface and protein surface features at site 15, 2–10, 14 and 17 influence more on PCA modification site determination.

### Features analysis of gain/loss of amino acids during evolution

There were 5 features of gain/loss of amino acids during evolution in the optimal feature set, which located at site 13, 10, 6, 18 and 20.

### Feature analysis of deviation of side chain carbon atom number

There were 5 features of deviation of side chain carbon atom number in the optimal feature set which located at site 8, 9, 13, 10 and 14, indicating that these sites may influence more on PCA modification site determination than other sites.

3.4 Directions for experimental validation

We investigated the top 20 features within the optimal feature set. Among which there are 14 features of PSSM conservation, 3 features of AAFactor, 2 features of secondary structures and 1 feature of deviation of side chain carbon atom number. The top 20 features indicate that conservation may play the main role in PCA modification site prediction.

## Discussion

**Figure 8:**
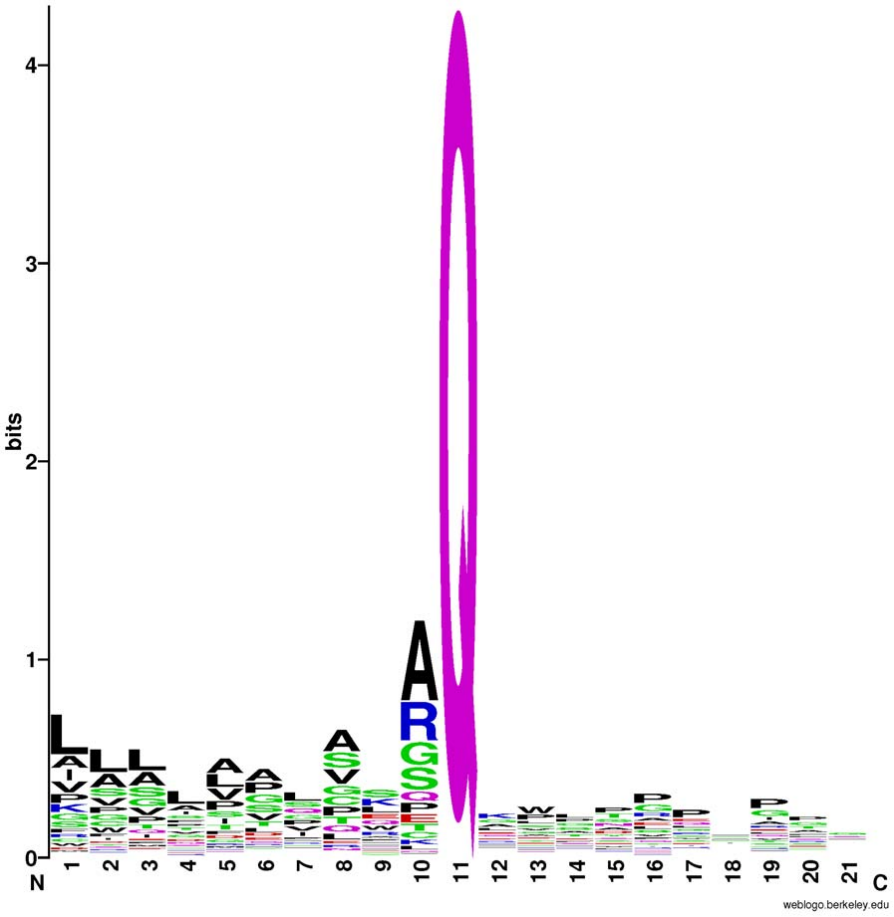
The sequence logo of 370 21-residue peptides [**49**]. In the upstream of Q site, sites 1, 2, 3, 5, 8 and 10 show some degree of conservation.

Due to biological importance, PTM sites and regions surrounding the sites often display some degree of conservation, such as phosphosites and their −5 and +5 residues [44]. Evolutionary analysis has also showed evidences of PTM positions' higher purifying selection in 70% of the phosphorylated proteins [45]. Obviously, PCA sites have the similar conservative property. Even they are so extremely conservative that nearly all of the sites are restricted to Glutamine (Q), implying the significance of Q in PCA modification. Mutations to these PTM sites or surrounding regions may cause diseases [46]. However, as Figure 8 shows that, there is no remarkable conservative pattern found in the downstream of the PCA modification site Q. In contrast, in the upstream of the Q site, there is some evidence to exemplify the conservation in the surrounding region. For example, sites 1, 2, 3, 5, 8 and 10 are more likely to be amino acids L, L, L, A and A . This is also to some extent consistent with our previous results. Of the optimized 224 features, based on which we could obtain the best prediction accuracy, more than a half are features of PSSM conservation score, indirectly demonstrating there is some conservation around PCA sites. In particular, for the prediction, it is not equally important for all positions to influence the accuracy. Our results displayed that site 10 played the most important role in the prediction, and then sites 1, 2, 3 and 5 also played more important role than the other remaining sites.

In order to improve the prediction accuracy of PCA modification sites, a wide range of different types and of sources of information have been combined. These include biophysical information like amino acid physicochemical and biochemical properties, gain/loss of amino acid during evolution, deviation of side chain carbon atom number, or structural information like secondary structure, solvent accessibility and disorder score. For example, it has been noted that many PTM sites have a relative tendency to occur in regions lacking secondary structure, such as regions of intrinsic disorder [47], [48]. One reason is that increased flexibility of intrinsic disorder regions allow them to fold, and therefore the amino acid side chains would fit into a modifying enzyme's catalytic site easily. Our results revealed that this feature of disorder score is not very important for the prediction, because we could find from Table 1, features of secondary structure played more important role. Nevertheless, Figure 5A indicated that three types of secondary structure features (helix, strand and other) influence the determination of PCA modification site nearly equally, a little inconsistent with others'. dbPTM and Pang et al. have both shown that PCA sites are more likely to be within coiled regions [17], [48]. This may be because our peptides are 21-residues long, much longer than theirs. In the article, Pang et al. have also manifested PCA modifications did not show any strong preferences for surface accessibility, completely compatible with ours. From Figure 6A, buried and exposed have the same influence for the prediction, suggesting this feature we selected was used correctly.

At present, our method is limited to PCA modification occurring in the internal region of the protein sequences. One pivotal reason is that, most (222/399) of the protein sequences containing PCA modified Q sites at the N-terminus have less than 21 residues. Consequently, for those at the N-terminal of sequences, it is necessary to establish a novel method, to predict the PCA modification which occurs at the amino-terminus of proteins or both by integrating our model.

In this study, we developed a method for the prediction of PCA modification sites using totally 727 features. Our method achieved an overall MCC of 78.12% using the optimal feature set (244 features). Further detailed feature analysis may provide clues for understanding the PCA modification mechanism. The selected optimal feature set, especially the top features may provide important clues for further experimental researches in this area.

## Supporting Information

Dataset S1 **Training dataset used in the study.**
(XLS)

Table S1 **IFS results.**
(XLS)

Table S2 **Optimal feature set.**
(XLS)
